# Phytochemical profiling and acute oral toxicity of *Suregada zanzibariensis* (Baill) root extract

**DOI:** 10.4314/ahs.v24i2.15

**Published:** 2024-06

**Authors:** Japhet Kimondo Josephat, Cyprian Beda Mpinda, Rose Justus Masalu

**Affiliations:** Department of Molecular Biology and Biotechnology, College of Natural and Applied Science, University of Dar Es salaam, P.O.Box 35179 Dar es Salaam, Tanzania

**Keywords:** Phytochemical profiling, acute oral toxicity, *Suregada zanzibariensis* (Baill) root extract

## Abstract

**Background:**

Traditional healers utilize the roots of *Suregada zanzibariensis* for managing diabetes mellitus. Therefore, evaluation of toxic properties of this plant is important.

**Objective:**

To evaluate acute oral toxicity of *S. zanzibariensis* root extract on Wistar rats and to screen phytochemical compounds of the EAESZ.

**Methods:**

GCMS analysis of the plant extracts were performed by using GCMS-2010 Shimadzu and mass spectra of the compounds found in the extract was matched with the data in the library of National Institute of Standards and Technology (NIST). Acute oral toxicity testing was carried by administering a single Distilled water extract (DWESZ) and EAESZ to four different groups of rats at dosage of 300mg/kg and 2000mg/kg in each extract to the separately group of rats

**Results:**

The GC-MS analysis of *S. zanzibariensis* roots extract revealed the presence of 10 major compounds. A higher single dose (2000mg/Kg) of EAESZ and DWESZ extract did not produce any sign of toxicity throughout 14 days of study, in terms of changes in behaviour or mortality in tested rats. No significant (p > 0.05) hematological, liver histological, biochemical changes were noticed between rats treated and control rats

**Conclusion:**

The results obtained suggest that the plant extract can be classified as non-toxic.

## Introduction

Worldwide, plants have been used as a source of medicine and 80–85% of populations rely on these medicinal plants to meet their primary health care needs 1. Literature suggest that, more than 80% of the population in sub-Saharan Africa relies on medicinal plants and Traditional Health Practitioners as the primary source of health care due to accessibility and cultural acceptance 2. A number of phytochemicals isolated from medicinal plants are directly used as drugs 2,3. For example, Metformin is an oral hypoglycemic agent isolated from medicinal plant *Galega officinalis* that was used historically in medieval Europe for the treatment of diabetes 3.

In Tanzania, people access a variety of resources to meet their healthcare needs, and at least 60 % of the population is estimated to use traditional medicines 4. Several medicinal plant are used traditionally to treat diabetes 5,6 reported that in Kilimanjaro, a northern region of Tanzania 77.1% of patients with diabetes mellitus use herbal medicines to treat this disease. One of the medicinal plants that has been used to manage diabetes mellitus in Kilosa is Suregada zanzibariensis' root 5. Furthermore, 7 reported that local communities in Tanzania use at least 62 different plant species to manage diabetes mellitus; however, 33 species have not been scientifically investigated including *Suregada zanzibariensis*. These examined plants' effects have been found to postpone the onset of diabetic symptoms and reverse several metabolic anomalies 8. Additionally, a significant proportion of medicinal plants have some level of toxicity. For instance, *Aspilia mossambiscensis* (Oliv.) Willd, *Caesalpinia bonducella* (L.) Flem, and *Phyllanthus amarus* have all shown a slight toxicity to animals 7.

The genus Suregada, also named Gelonium, belongs to the tribe of Gelonieae, subfamily of Crotonoideae in the Euphorbiaceae family. About 30 species make up the genus Suregada, 22 of those found in Africa 9. *Suregada zanzibariensis* Baill is widely spread in the east coastal belt of Africa where by, in Tanzania is mostly found in Ruvuma, Bagamoyo, Zanzibar, and Morogoro 10,11. *Suregada zanzibariensis* also called mdimu msitu in Tanzania, is a timeless shrub or a small tree with horizontal branches that grows up to 0.5–10 m tall, and occurs usually on sandy soils in woodland, riverine forest, coastal forest and in salt marshes, from sea-level up to 1200 m altitude 5. The root and stem bark extracts of *Suregada zanzibariensis* are used in Tanzania to treat ancylostamiasis and a tea from the roots is drunk to treat gonorrhoea, stomach ache, chest pain, hernia, pneumonia and snake bites and the leaves are used to treat skin infections 12. On other hand it has been reported that traditional healers are using the roots decoction of *Suregada zanzibariensis* to treat and manage diabetes mellitus 5. Despite its widespread use, there have not been any research on its toxicological effect. The aim of this study is to investigate the acute toxicity and phytochemical profiling *Suregada zanzibariensis* in

## Material and Methods

### Description of the Study Area and Sample Collection

Fresh *S. zanzibariensis* root weighing 2 kg were collected in January 2022 from a bushy area close to a damp site at the University of Dar es Salaam Mwalimu JK Nyerere campus. The collection site has an altitude of 104m, GPS 37m 521958 utm 9250774. A botanist performed preliminary plant identification in the field. The plant materils were certified at the Herbarium of the Department of Botany, University of Dar-es-Salaam, where voucher specimens were coded and given a voucher specimen number FMM 4140 and later on deposited, using fresh leaves and roots of *S. zanzibariensis*.

### Chemical, Consumables, and Biological materials

In this research study, several crucial materials were utilized, including, the chemical components such as ethyl acetate manufactured by Loba Chemie PVT. LTD and xylene. These substances played essential roles in various processes throughout the research. To facilitate the necessary measurements and analyses, a range of consumable items was employed including specific test kits for aspartate aminotransferase (AST) and alanine aminotransferase (ALT) to assess liver function. Additionally, blood collecting tubes were used to obtain blood samples for hematology examination. For histology of liver tissue and pancreas, glass slides and microtome blades were indispensable tools. Moreover, the research relied heavily on biological materials, particularly rats, which served as the primary subjects for the investigation. Furthermore, the roots of *S. zanzibariensis* was used to obtain EAESZ and AQUESZ. Also, the equipments used are rotary evaporator Model number RE-501 manufacture by Labs Nova, USA. Manifold freeze dryer, model number LGJ^10^, made by Movel Scientific Instrument Co., Ltd USA.

### Experimental Animals

The care and handling of animals in this experiment were according to internationally accepted ethical guidelines for use of laboratory animals 15. Eighteen male rats with weights ranging from 85 to 100g and eight weeks of age were collected from the Sokoine University of Agriculture (SUA)-Morogoro laboratories and raised in the Zoology Laboratory, University of Dar es Salaam. The animals were kept in cages (3 rats per cage) at room temperature with a 12 h/12 h light/dark cycle, according to^15^,^16^. Throughout the experiment, animals were provided standard pellet (food) and water except fasting period.

### Plant Sample and Preprocessing

Fresh roots were properly washed with tap water, rinsed with distilled water and left to dry under shade in a well-ventilated area. After 21 days dry samples were macerated and stored in airtight containers at room temperature for experimental use. Analytical grade solvents; ethyl acetate 99.5% purity and distilled water were used for extraction. 500 g of powdered *S. zanzibariensis* roots were weighed, then each soaked in 1.5 L of distilled water and 1.5 L of ethyl acetate at room temperature. The mixture was shaken every 12 hours for two days, then filtered using Whatman No. 1 filter paper with pore size of 11 um. The ethyl acetate extract was dried using rotary evaporator while the aqueous extract was dried using a manifold freeze dryer according to 13. Obtained crude extracts were stored at 4°C until required for experiments.

### Phytochemical Profiling

Analysis of phytochemicals in the *S. zanzibariensis* root extracts was done by Gas Chromatography- Mass Spectrometry according to (14). GC-MS was recorded in a GCMS-2010 Shimadzu instrument operating in Electron Ionization (EI) mode (MS) at 70ev, and Flame Ionization Detector (FID) for GC. A Restek-5MS column (30m x 0.25mm x 0.25µm) was used. The oven temperature program was 90°C to 28°C, and held at 90°C for two minutes. The temperature was increased to 280°C for 10 minutes (hold time) at the rate of 15°C per minute. The injection temperature was 250°C with split injection mode. The flow rate of carrier gas helium was 1.21ml min-1. The ion source temperature and interface temperature in MS was 230°C and 300°C , respectively. The identification of phytochemicals in the extracts was done by scan method which involves the use of Mass Spectral Library and Search Software (NIST 11). Quantification of phytochemicals in the extracts was done using Peak Integration method (area normalisation) whereby ion allowance was 20%, target ion and other five quantitation ions were used on quantitative analysis. A 10 uL of the sample was dissolved in dichloromethane to make 1mL and injected in GC MS then in the warm inlet, the solution evaporated and turned into gas. The sample was transported across the column by the mobile phase (helium gas). Depending on their chemistry, various elements in the sample interacted with the stationary phase of the column in various ways. As a result, they separate from one another as they go through the column at various speeds. After that, the separated compounds exited the column one at a time and entered a detector (mass spectrometer). The results were reported as percentage compositions derived from peak area of all scanned compounds in the extracts.

### Toxicity Assay

#### Acute Oral Toxicity Tests of the Suregada zanzibariensis Extract

An acute oral toxicity study was carried out according to 17 guidelines 423. The detailed experimental treatment schedule is shown in Table 1. Different single doses of *S. zanzibariensis* (300, 2000 mg/kg) were suspended in 5% of tween 80 and administered to SZE-treated group (n = 3), by using oral gavage feeding technique. There was also control group (n = 3) administered 5% of tween 80. The signs of toxicity (lacrimation, hair erection, convulsion, coma, and death) due to these doses were observed every day for 14 days. Additionally, rats' body weights were measured at day 0 (BWo) and then every seven days until day 14. The following formula was used to determine the percent change in body weight as a function of the rats' starting body weight on day zero:


$$Percentage\;change\;in\;body\;weight=\frac{BWn-BWo}{BWo}100$$

Where BWn = Body weight of rats on respective days and BWo = Body weight of rats the first day.

**Table 1:** Experimental treatment schedule for OECD 423 acute toxicity studies of *S. zanzibariensis* extract (SZE)

S/N	Experiment treatment schedule
1	Group I control (5% of tween 80, bw.; n = 6)
2	Group II (SZE 300 mg/kg bw.; n = 3, as performed twice)
3	Group III (SZE 2000 mg/kg, bw.; n = 3, as performed twice)

### Hematology Analysis

On the 15^th^ day of the acute oral toxicity test, all animals were fasted for 12 hours and then anaesthetized by placing them in a desiccator which contained cotton wound wetted with chloroform 99.8% purity and then sacrificed. Blood samples (3 ml) were withdrawn by cardiac puncture and collected immediately into tubes containing EDTA for analysis of hematological parameters. Hematological analysis for white blood cell count, red blood cell count (RBC), platelet count, hematocrit, hemoglobin (Hb), mean corpuscular volume (MCV), mean corpuscular hemoglobin (MCH), mean corpuscular hemoglobin concentration(MCHC), neutrophils, eosinophils, monocytes, basophiles, total platelet count and lymphocytes was done using a hematology analyser Sysmex NX550 Auto Hematology Analyzer, KOBE, JAPAN.

### Biochemical Analysis

In addition, all animals were anaesthetized and sacrificed after a 15-day acute oral toxicity assessment. Blood was drawn from the rat via a cardiac puncture and immediately placed in tubes containing no EDTA. The blood was then placed into Eppendorf tubes (5.0 mL) and centrifuged at 4000 r/min for 10 minutes at 4°C to extract serum, which was kept at -80°C. To measure Aspartate Aminotransferase (AST) and Alanine Aminotransferase (ALT) in serum, commercial kits from Erba Mannheim, Germany, known as Erba Aspartate Aminotransferase (AST) Test Kit and Alanine Aminotransferase (ALT) Test Kit, were utilized. AST or ALT substrate (R1) and AST or ALT coenzyme (R2) were combined in a 4:1 ratio to create the working reagent (15 ml) for the AST and ALT tests. After that, 0.1 ml of a sample and 1 ml of the working reagent were combined and incubated at room temperature for one minute. When L-aspartate or L-alanine and 2-oxoglutarate are combined, AST or ALT in the sample catalyzes the transfer of an amino group, forming oxaloacetate and L-glutamate. In the presence of malate dehydrogenase (MDH), the oxaloacetate then combines with NADH to produce NAD+. The catalytic AST or ALT activity directly correlates with the rate of NADH oxidation. The reduction in absorbance at 340 nm caused by the oxidation of NADH to NAD is measured to determine out. The absorbance changes (ΔA) exactly after 1, 2 and 3 minutes was measured using a UV-Vis spectrophotometer (Libra S50PC, 118436). The level of AST or ALT in a serum was calculated using the following formula.


$$\begin{array}{cc} AST\;or\;ALT=F\frac{\Delta A}{Min}, & \text{Factor}(F) \end{array}=952$$

### Histopathology Analysis for Liver

For histologic studies, the liver was taken and fixed in 10% neutral buffered formalin. The fixed samples were dehydrated in ascending series of ethanol, cleared in xylene, and embedded in paraffin wax. Sections of 5-µ thickness were prepared using a microtome, section was fixed in the slide and stained with hematoxylin and eosin (H+E), and studied under a light microscope. The sections were evaluated based on the severity of the pathological changes.^18^

### Ethical clearance

The University of Dar es Salaam ethical committee granted permission for the study to be carried out and to utilize laboratory animals.

### Statistical Analysis

The statistical test that was used in this study was two sample tests. Data was expressed as a mean ± standard deviation; differences among treatment group means was assessed to see if there is significant difference between the mean of treatment group and the controlled group. Data was statistically evaluated the confidence level P < 0.05

## Result

### GC-MS Analysis

A total of ten phytochemical compounds from an ethyl acetate extract of *S. zanzibariensis* were identified by GC-MS analysis as shown in Table 2.

**Table 2:** Phytochemical compounds detected in ethyl acetate extract of *S. zanzibariensis* root by GC-MS

Name of compound	Chemical structure	RT	m/z	Cone	Area
3-Tetradecene, (Z)–	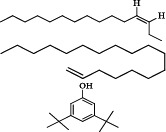	4.565	41.05	3.38479	65866
3-Hexadecene, (Z)-		5.971	43.1	6.9427	135101
Phenol, 3,5-bis(1,1-dimethylethyl)-		6.745	191.2	26.19964	509830
1-Octadecene	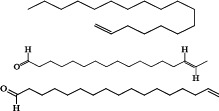	7.129	43.1	9.07211	176538
E-15-Heptadecenal		8.128	43.1	9.84279	191535
n-Heptadecanol-1		9.02	55.1	8.93248	173821
10-Octadecenoic acid, methyl ester	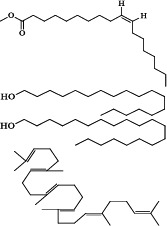	9.48	55.1	6.68478	130082
n-Nonadecanol-1		9.83	43.1	7.27349	141538
1-Heneicosanol		10.566	57.1	5.20992	73186
Squalene		12.107	69.1	16.45732	217531

### Acute Oral Toxicity Analysis

#### Effect of *S. zanzibariensis* Extract on Body Weight

**Figure 1:**
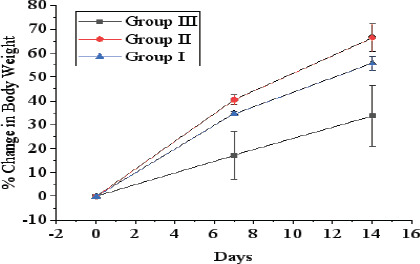
Percent change in body weight of normal and EAESZ treated rats. Each error bar represents a mean ± standard deviation

**Figure 2:**
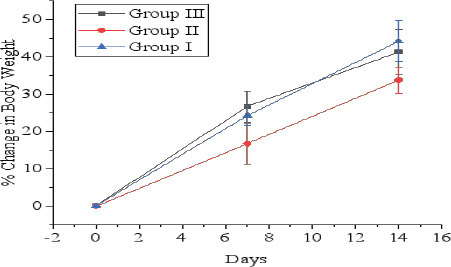
Percent change in body weight of normal and DWESZ treated rats. Each error bar represents a mean ± standard deviation

**Figure 3:**
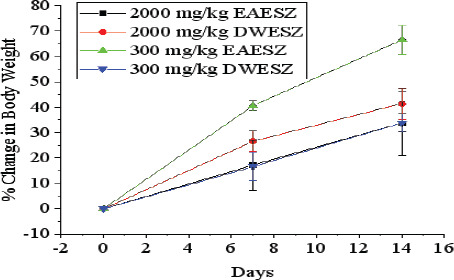
Percent change in body weight in comparison of two doses of 300 mg/kg and 2000 mg/kg of both EAESZ and DWESZ of normal and DWESZ. Each error bar represents a mean ± standard deviation

Body weight was measured on 1st day before administration of a dose. On 7^th^ and 14^th^ day, changes in body weight are depicted in Figures 1 and 2. It was observed that in all groups of animals (Groups I, II, and III) administered DWESZ at single dose of 300 mg/kg and 2000 mg/kg body weight for group II and III respectively, there was no significance increase in body weight as compared to group I administered 5% tween 80 (p > 0.05). Also, there was no significance increase in body weight for group II administered single dose of 300 mg/kg body weight of EAESZ on 7^th^ and 14^th^ day (p > 0.05), whereas there was significant increase in body weight for group III on 7^th^ day (p < 0.05) and no significant increase on body weight on day 14^th^ as compared to group I administered 5% tween 80 (p > 0.05). Additionally, animals given single doses of 300 mg/kg and 2000 mg/kg of EAESZ and DWESZ each did not significantly show any difference in increasing their body weight (p > 0.05) as shown in figure. 3

#### Effect of EAESZ and DWESZ on Biochemical Parameters

Tables 3 and 4 illustrate the effect of a single dosage of EAESZ and DWESZ on serum biochemical parameters. Table 3 shows that there was no statistically significant difference (p > 0.05) between the control and EAESZ administered rats, while there was a significant difference in ALT levels between group III treated rats and control group (p < 0.05). Table 4 demonstrates no significant difference in AST and ALT level (p > 0.05) between the control and DWESZ treated rats of group II and group III

**Table 3:** Effect of EAESZ (Dose in mg/kg body weight) on biochemical parameters in acute oral toxicity study. Values are expressed as mean ± standard deviation (n = 3) for each group

Experimental groups	Biochemical parameter			

	ALT (U/L)	ALT (U/L) Reference range(Hasan et al. 2018)	AST (U/L)	AST (U/L) Reference range (Hasan et al. 2018)
Group I (Control 5% Tween 80)	21.89±0.83	10- 40	68.18±18.17	50- 150
Group II (300 mg/kg)	22.05±2.62	10- 40	73.77±12.14	50- 150
Group III (2000 mg/kg)	17.29±4.39	10- 40	68.69±14.62	50- 150

**Table 4:** Effect of DWESZ (Dose in mg/kg body weight) on biochemical parameters in acute oral toxicity study. Values are expressed as mean ± standard deviation (n = 3) for each group

Experimental groups	Biochemical parameter			

	ALT (U/L)	ALT (U/L) Reference range(Hasan et al. 2018)	AST (U/L)	AST (U/L) Reference range (Hasan et al. 2018)
Group I (Control 5% Tween 80)	17.91±4.08	10- 40	57.63±5.41	50- 150
Group II (300 mg/kg)	18.49±2.58	10- 40	57.14±2.10	50- 150
Group III (2000 mg/kg)	17.16±4.28	10- 40	58.66±2.50	50- 150

#### Effect of EAESZ and DWESZ on Hematological Parameters

After 14 days of administration of a single dose of *S. zanzibariensis* roots extract, there was no significant difference (p > 0.05) in neutrophil, monocytes, platelet count, basophils, hematocrit, mean corpuscular volume (MCV), and eosnophils between the control group treated with a single dose of 5% tween 80 and experimental groups given 300 mg/kg and 2000 mg/kg of *S. zanzibariensis* extract in group II and III respectively while there was a significant difference (p < 0.05) in red blood cell (RBCs), lymphocytes, mean corpuscular hemoglobin (MCH), mean corpuscular hemoglobin concentration (MCHC) and hemoglobin (Hb) between the experimental group administered with single dose of EAESZ groups and control group as shown in Table 5.

**Table 5:** Effect of ethyl acetate extract of *S. zanzibariensis* roots (Dose in mg/kg body weight) on hematological parameters in acute oral toxicity study. Values are expressed as mean ± standard deviation (n = 3 for each group)

PARAMETER	CONTROL	2000 mg/kg	300 mg/kg	REFERENCE (Filho et al., 2017, Santos et al. 2013.)
Red blood cell (RBC)	6.96±0.38	6.22.68±0.248	5.9±0.537	5.2 – 10.4
Haemoglobin (Hb) g/dl	11.36±2.91	12.36±1.28	11.53±1.9	11.1 – 14.8
Haematocrit L/L	0.4±0.028	0.33±0.042	0.34±0.028	0.321–0.465
Mean corpuscular volume (MCV) Fl	57.47±0.05	57.73±0.59	57.3±0.51	44.2 – 58.5
Mean cell haemoglobin (MCH) pg	17.23±2.62	18.42±0.37	19.06±1.45	14.0 – 18.7
Mean cell haemoglobin concentration MCHC (g/dl)	32.6±0	33.2±1.91	33.17±2.65	28.4 – 38.5
RDW %	15.57±0.72	16.0±0.75	15.83±1.04	11 – 16
Neutrophil (%)	15.38±1.68	15.3±4.32	14.53±2.02	10 – 23
Lymphocytes (%)	66.88±0.65	68.22±9.68	70.9±4.15	74 – 90
Monocytes (%)	3.1±2.0	1.96±1.05	2.1±0.43	0 – 5
Eosinophils (%)	2.67±1.06	2.63±1.12	2.4±0.82	0 – 3
Basophils (%)	12.46±2.44	12.38±2.67	11.13±4.65	0 – 1
Platelets	604.33±242.6	577.33±234.5	572.33±242.14	315 – 758

However, as shown in table 6 there was no significant difference in white blood cell count, red blood cell count (RBC), platelet count, hematocrit, hemoglobin (Hb), mean corpuscular volume (MCV), mean corpuscular hemoglobin (MCH), and mean corpuscular hemoglobin concentration (MCHC) and lymphocytes between the control and experimental groups given a single dose of 300 mg/kg and 2000 mg/kg of aqueous extract of *S. zanzibariensis* (p > 0.05). But there was a significant difference in basophil levels between the control group and the treated group at a dose of 300 mg/kg body weight (p < 0.05). When comparing the hematological results of this study to the reference range, it is clear that all of the results were within normal limits, with the exception of basophils, which were higher while lymphocytes were lower in groups I, II and III given 5% tween 80, 300 mg/kg and 2000 mg/kg body weight of ethyl acetate extract of *S. zanzibariensis*.

#### Effect of EAESZ and DWESZ on Liver Cells

**Figure 4:**
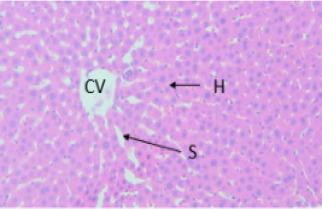
Control animal treated with 5% tween 80, displaying normal arrangement hepatocytes (H), sinusoids (S) and central veins (CV) unaffected, uniform sized cells were present. Haematoxylin and Eosin stained liver tissue, 40X

**Figure 5:**
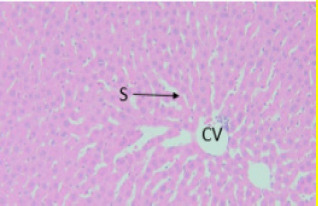
Treated rat with EAESZ at dose of 300 mg/kg. Histopathology displaying exemplary setup of hepatocytes, clearly visible hepatic sinusoids (S) and central veins (CV) unaffected. Haematoxylin and Eosin stained liver tissue, 40X

**Figure 6:**
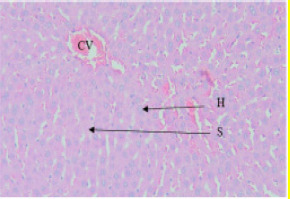
Treated rat with EAESZ at dose of 2000 mg/kg. Histopathology displaying normal arrangement of hepatocytes (H), absent clearly visible hepatic sinusoids (S) and clearly visible central veins (CV) unaffected. Each cell has a consistent size. Haematoxylin and Eosin stained liver tissue, 40X

**Figure 7:**
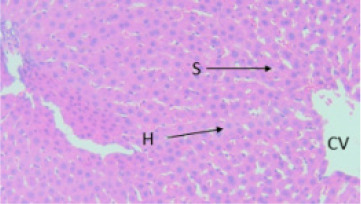
Treated with DWESZ at dose of 300 mg/kg. Histopathology displaying hepatocytes (H), sinusoids (S) and central veins (CV). Each cell has a consistent size. Liver architecture unaffected. Haematoxylin and Eosin stained liver tissue, 40X

**Figure 8:**
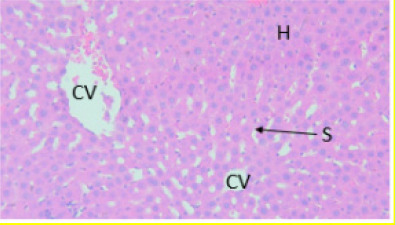
Treated with DWESZ at dose of 2000 mg/kg. Histopathology displaying normal arrangement of hepatocytes (H), without obvious sinusoids (S) and clearly visible central veins (CV). Each cell has a consistent size. Liver architecture unaffected. Haematoxylin and Eosin stained liver tissue, 40X

Light microscopic observation with the doses of 300 mg/kg and 2000 mg/kg of both EAESZ and DWESZ (Figure. 5, 6, 7 and 8) which administered once by oral gavage showed no marked histopathological changes on the livers histoarchitecture of rats as compared to the control group (Figure. 4). The liver histology of the control rats and treated rats showed normal microstructural characteristic such as hepatic lobules, central vein lined by endothelial cells, radiating hepatic cells, Kupffer cells and hepatic sinus

## Discussion

Non-nutritive plant compounds with disease-preventive or protective characteristics are known as phytochemicals 19. Phytochemicals have been known to have a wide range of actions including antimicrobial, antidiarrheal, antibacterial, and anti-inflammatory properties. For example, antidiarrheal action is suggested to be due to tannin and flavonoid 14,20. Tannin, terpenoid, alkaloid, steroid, and flavonoid phytochemicals have anti-inflammatory properties 21. Glycoside, alkaloid, tannin and flavonoid have hypoglycemic properties and are useful in lowering blood pressure and protecting the heart 22. Furthermore, flavonoids are antioxidants and free radical scavengers that protect cells against oxidative cell damage, have potent antitumor action, and protect cells from carcinogenesis at all stages. Flavonoid also have anti-allergic and anti-inflammatory properties 21,23. The GC-MS analysis of S. zanzibariensis roots extract revealed the presence of 10 major compounds which are Phenol, 3,5-bis(1,1-dimethylethyl)-, Squalene, 1-Heneicosanol, n-Nonadecanol-1, 10-Octadecenoic acid, methyl ester, n-Heptadecanol-1, E-15-Heptadecenal, 1-Octadecene and 3-Tetradecene, (Z)-. These compounds were in different concentration as shown in table 2.

Among identified phytochemical is squalene. Squalene is a triterpenoid that has a role in the biosynthesis of sterols in plants 24. Squalene is a key intermediate component in the production of eukaryotic sterols and is found in practically all cells since it is a sterol/cholesterol precursor molecule 24. About 60–85% of dietary squalene is absorbed and transported through the serum, primarily with VLDL, before being dispersed to various organs. Only a small portion of the vitamin squalene is turned to cholesterol. Squalene levels in the blood are safe, helpful, and have chemopreventive and hypocholesterolemic effects^25^. Squalene also has antioxidant properties; according to^26^, Squalene has an antioxidant impact against isoproterenol-induced myocardial infarction by inhibiting lipid peroxidation. Squalene's cardioprotective properties may be owing to its antioxidant and membrane stabilizing properties 26.

Also, GC-MS analysis of *S. zanzibariensis* extract showed the presence of Phenol, 3,5-bis(1,1-dimethylethyl)-. This compound is responsible for inhibiting amylase and glucosidase, according to 27. Polyphenols have the ability to chelate enzymes, leading them to precipitate and lose their biological activity as a result of structural changes^27^. On other hand, n-Nonadecanol-1 has also been reported to exhibit anti-microbial and cytotoxic activities 28. Furthermore, E-15-Heptadecenal and 1-Heneicosanol are antifungal and have been shown to inhibit Trichophyton mentagrophytes completely at a concentration of one percent 29. Antibacterial, antifungal, anticancer, anti-inflammatory, and antioxidant activities are also found in E-15-Heptadecenal 43.

During the 14^th^ -day trial, a maximum dose of 2000 mg/kg body weight. of both (aqueous and ethyl acetate extract) caused no signs of toxicity or fatality. All animals were confirmed to be healthy throughout the 14-day period, with no changes in their behavioral characteristics. As a result, it's reasonable to assume that its oral LD50 value is more than 2000 mg/kg of body weight.

Decreases in body weight can be caused by the toxic effects of plant components, which can lead to a fall in appetite and a reduction in animal caloric intake 30. From day 1 to 14, all groups in the acute toxicity research of AQUESZ, as well as groups I and II in the acute toxicity investigation of EAESZ, had increase body weights. According to 31 increases in body weight are accompanied with fat formation and physiological adaption responses to plant extracts. The normal weight gain of rats in this study treated with the extracts (AQUESZ and EAESZ) at all doses is evidence that the extracts did not interfere with normal metabolism and appetite stability, which would have caused an decrease in food and water intake and decreased body 31.

Furthermore, compared to groups I and II, which gained weight at rates of 40.6 percent and 34.7 percent, respectively, group III gained only a small amount of weight (17.3 percent) during the first week after receiving a single dose of EAESZ; therefore, there was a significant difference between group I (Control) and treated group III (p < 0.05). This might be because rats in group III received higher doses of a plant extract that contains possible hypoglycemic agents such phenol, 3,5-bis(1,1-dimethylethyl), and squalene 24. Due to a lack of glucose, hypoglycemic compounds in plant extract increase effective oxidation of fatty acids into fuel by activating adenosine 5 monophosphate kinase (AMPK), an enzyme that plays a key role in cell energy and metabolism ((Ravi et al. 2016). AMPK facilitate fatty acid oxidation in cells since may be there was inhibition of glucose absorption. Hence fats/fatty acids are used as a source of energy when glucose is scarce. Fat cells release their fat contents to maintain energy balance when there is a constant need for energy in the absence or scarcity of glucose. This increased fatty acid oxidation eventually leads to weight loss 32.

When inflammation or disease processes impact liver cell activity, both serum aspartate aminotransferase (AST) and alanine aminotransferase (ALT) levels rise; nevertheless, ALT is the more liver-specific enzyme (33). The enzyme aspartate aminotransferase (AST) is found in a variety of tissues, including the liver, heart, muscle, and kidney (Odiegwu 2021). In illnesses affecting these tissues, elevated AST serum levels are reported. Furthermore, ALT activity elevations last longer than AST activity elevations because AST levels rise early in the course of liver injury and have a limited lifetime^34^.

There was no significant difference in ALT and AST levels between the control and rats given either aqueous or an ethyl acetate extract of *S. zanzibariensis* roots. Since a rise in ALT and AST levels is usually a characteristic finding in disease and inflammation that impacts liver cells, no significant alternation after administration of aqueous and ethyl acetate extract of *S. zanzibariensis* roots suggests that there was no possible inflammation occurred at the doses of 300 mg/kg of both aqueous and ethyl acetate and dose 2000 mg/kg of ethyl acetate extract tested^30^,^35^. Furthermore, after a single dose of both aqueous and ethyl acetate extract of *S. zanzibariensis* roots, the levels of ALT and AST were within the normal ranges of 10 to 40 and 50 to 150 IU/L for ALT and AST, respectively. This indicates that the extract did not cause tissue or liver cell damage^33^.

Hematologic variables are highly sensitive biomarkers that are commonly associated with physiological responses to toxic substances^36^. When hematological results of groups I, II, and III given a single dose of 5 percent tween 80, 300 mg/kg, and 2000 mg/kg of both ethyl acetate and aqueous extract *S. zanzibariensis* roots were compared to the reference range, white blood cell (WBC) levels were found to be within normal limits. In all experimental groups, however, the percentage of basophils was much higher. In rats administered 5 percent tween 80, 300 mg/kg, and 2000 mg/kg BW of the EAESZ, lymphocytes significantly decreased (p < 0.05).

The higher percentage of basophils in groups I, II, and III after receiving a single dosage of 5% tween 80, 300 mg/kg and 2000 mg/kg of both EAESZ and AQUESZ respectively, could be attributed to a number of reasons interfering with the hematological parameters' results. The approach utilized in blood collection, as well as the collection site, may have contributed to the greater percentage of basophils in this study^37^. This is because it was anticipated that if the extract was an allergen or produced inflammation, the level of basophils, eosinophils, and lymphocytes in the bloodstream would rise over the usual range^38^,^39^. However, the percentage of basophils in this study was higher than usual, whereas the percentage of eosinophils was normal, and lymphocytes were low. These findings indicate that the root extract of *S. zanzibariensis* does not cause an allergic reaction or inflammation in the peripheral tissue.

Lymphocytes and neutrophils often increase their levels in response to a hazardous environment^30^. Leucocytes were unaffected by this study since they were within normal limits. However, lymphocytes were low in all experimental groups given a single dosage of 5% tween 80, 300 mg/kg, or 2000 mg/kg of ethyl acetate extract of S. zanzibariensis root, whereas neutrophils were normal. The presence of anticancer phytochemicals in the extract that promote apoptosis could be to account for the low lymphocyte count (40). This indicates that the *S. zanzibariensis* ethyl acetate extract causes no tissue harm in the animals.

There was a significant difference in RBC, hemoglobin (Hb), mean corpuscular hemoglobin (MCH), and mean corpuscular hemoglobin concentration (MCHC) between the control group treated with a single dose of 5% tween 80 and the experimental groups given single doses of 300 mg/kg and 2000 mg/kg of ethyl acetate extract *S. zanzibariensis* roots in groups II and III, respectively (p < 0.05). Also, there was no significant difference in MCV and hematocrit between groups I (control) and II and III (p > 0.05).

Increases in RBC count, hemoglobin (Hb), mean corpuscular hemoglobin (MCH), and mean corpuscular hemoglobin concentration (MCHC) in groups II and III after administration of a single dose of 300 mg/kg and 2000 mg/kg of EAESZ, respectively, could be due to components in crude extract of *S. zanzibariensis* that stimulate the kidney directly to cause formation and secretion 41.

Because the rats were in normal physiological condition, the mean corpuscular volume (MCV) was normal in all experimental groups. According to 42, the deforming force of red blood cells (RBC) is primarily exerted from outside the cell by fluid shear stress and from within the cell by changes in the osmotic milieu under physiological settings. Furthermore, the lack of a significant difference in MCV between group I (control) and groups II and III, as well as MCV in the normal range, could be due to RBC deformability, which improves flow characteristics and protects against cell rupture 42. Significant increases in red blood cell count, haemoglobin concentration, and mean corpuscular hemoglobin (MCH) and mean corpuscular hemoglobin concentration (MCHC) were seen in this study as a result of the extract's direct impact on the haematopoietic systems. This shows that the crude extract of *S. zanzibariensis* root is safe.

Liver is the main organ responsible for metabolism and detoxication, It is also involved in hematopoiesis 43. Liver is involved in the metabolism of toxic compounds taken during eating. Change in histoarchitecture of livers cell is normally caused by cytotoxic compound eaten or increase in the oxidative stress of the cell and decrease in the antioxidant agents 18. In a present study, rats treated with all the doses of EAESZ and DWESZ did not show any morphological changes in the liver cells. That is none of pathological symptoms of liver were observed in all the rats investigated, hence EAESZ and DWESZ did not affect the liver. This might be verified by the results of biochemical analysis of liver enzymes such as ALT and AST in serum and hematological analysis which all were in a normal level hence indicate that liver was not be affected by the extracts 30,35,37.

## Conclusion

In this study, oral administration of the extracts (EAESZ and DWESZ) at doses of 300 and 2000 mg/kg did not result in any appreciable alterations in behavior, hematological parameters, liver histology, or liver enzyme levels. This shows that under the conditions that was observed in rats, the extracts are not toxic. Also, GC–MS analyses was performed to explore the phytochemical constituent's profile of EAESZ and revealed the presence of 10 major compounds which are known to posses many important activities like antioxidant, hypoglycemic, hypocholesterolemic and antimicrobial activities. Hence, *S. zanzibariensis* as a medicinal plant might have a wide range in manage and treating different disease. From the present study, further pharmacological studies at large scale is needed to confirm the exact compounds responsible for health benefits like hypocholesterolemic and antihyperglycemic.
